# Synergistic Reinforcement of Polyvinyl Alcohol Nanocomposites by Calcined Eggshell and Carbon Nanotubes

**DOI:** 10.3390/polym18091033

**Published:** 2026-04-24

**Authors:** Soo-Tueen Bee, Lee Tin Sin, Sin-Yee Yeoh

**Affiliations:** 1 Department of Mechanical and Material Engineering, Lee Kong Chian Faculty of Engineering and Science, Universiti Tunku Abdul Rahman, Jalan Sungai Long, Bandar Sungai Long, Cheras, Kajang 43000, Selangor, Malaysia; 2 Department of Chemical Engineering, Lee Kong Chian Faculty of Engineering and Science, Universiti Tunku Abdul Rahman, Jalan Sungai Long, Bandar Sungai Long, Cheras, Kajang 43000, Selangor, Malaysia; leets@utar.edu.my

**Keywords:** polyvinyl alcohol, eggshells, carbon nanotubes, composites, calcination, biodegradable

## Abstract

This study investigated the impact of incorporating calcined eggshell and carbon nanotube (CNT) on the properties of polyvinyl alcohol (PVOH) blends. Prior to solution casting, eggshell waste underwent a calcination process and then the samples were prepared via solution cast method. Mechanical properties study revealed a significant enhancement in tensile strength and elongation at break with increasing loads of calcined eggshell and CNT. Higher tensile strength was observed with increasing CNT loading for PVOH blends added with 1 phr and 3 phr calcined eggshell, owing to the reinforcing role of CNT in the composite matrix. In contrast, the tensile strength at 0.3 phr CNT is lower than at 0.2 phr CNT due to CNT agglomeration, which weakens the interfacial adhesion with the PVOH matrix and hinders effective stress transfer during deformation. SEM images depicted well-dispersion and interaction effect of calcined eggshell particles and CNT particles at low loading levels. The good interaction effect between calcined eggshell and PVOH matrix (which both exhibit hydrophilic behaviour) is mainly attributed to the presence of hydrogen bonding in the polymer matrix, as proven in FTIR analysis. XRD analysis revealed significant peaks in the 2θ range of 19° to 21°, suggesting that increased amounts of calcined eggshells influenced the crystallite size of the original PVOH matrix. In summary, the addition of calcined eggshell and CNT at low loading levels markedly enhanced the mechanical, physical, and thermal properties of the composite material.

## 1. Introduction

Over the past several decades, increasing attention has been devoted to biodegradable polymers, which are broadly classified into two principal categories: natural polymers and synthetic polymers. While synthetic polymers are derived from non-renewable petroleum sources, natural polymers are extracted from renewable resources [1]. In recent years, there has been a notable shift from the use of biostable materials to biodegradable materials for biomedical and related applications, particularly in areas such as temporary implants, drug delivery systems, tissue engineering scaffolds, wound dressings, and resorbable medical devices. Biodegradable materials are utilized to augment, evaluate, replace, and treat various organs or functions of the body. Biocompatibility is a crucial qualification for biomaterials that refers to their ability to perform appropriately within the body. Future trends suggest that many developing therapeutic devices, such as scaffolds and temporary therapeutics, will transition to biodegradable devices which facilitate tissue regeneration and self-repair. This transition is primarily motivated by long-term biocompatibility issues associated with biostable materials, including technical and ethical concerns related to revision surgeries [2]. The bioactivity of natural biodegradable polymers offers both advantages and disadvantages. While these polymers provide receptor-binding ligands to cells and support natural remodelling processes, they also pose risks such as disease transmission and strong immunogenic responses associated with most polymers [3].

Compared to synthetic biodegradable polymers, natural counterparts exhibit lower mechanical strength, repellent characteristics, and less predictable degradation rates [4]. Synthetic biodegradable polymers are favoured in biomedical applications due to their biologically inert properties, ability to provide satisfactory mechanical properties, and automatic degradation within the body over time. Moreover, synthetic polymers can be modified to meet specific requirements in the biomedical field, potentially reducing the need for additional surgeries and cutting medical costs. Polyvinyl alcohol (PVOH) is one of the common synthesis biodegradable polymers, which can be gauged by monitoring the degree of hydrolysis. Notably, PVOH chains possess a hydrophilic nature due to the presence of hydroxyl groups on carbon atoms, resulting in their water-soluble behaviour [5]. Upon formation, PVOH exhibits semi-crystalline characteristics and is known for its biodegradability, chemical resistance, and water solubility. Its widespread application in biomedical fields is attributed to its physical properties, which render it biocompatible with human tissue [6]. Biocompatible PVOH structures facilitate good cell adhesion and protein molecule absorption while posing no toxic effects on the human body [7]. It can be either physically entangled with or chemically bound to nanoparticle surfaces to enhance its properties.

Carbon nanotubes (CNTs) offer unique characteristics such as light weight, high aspect ratios, flexibility, strength, and toughness. They have been extensively utilized as reinforcements in various materials, including ceramics, metals, and polymers. The introduction of CNTs into polymer matrices could modify and improve the thermal, mechanical, and electrical properties and reduced costs of polymer matrix composites, further enhancing their contribution [8]. Due to these properties, carbon nanotubes are widely used in biomedical applications. In research conducted by Nadeem et al. [9], the authors performed and prepared carbon nanotubes functionalized with single-stranded DNA for the application of biological sensing and imaging. In addition, Sahithi et al. [10] prepared carbon nanotubes incorporating chitosan–epoxy nanocomposites targeted for the use in the biomedical application such as implants and medical devices. However, the desired properties of CNTs-based polymer composites depend heavily on dispersion and interfacial bonding between CNTs and the polymer matrix [11]. Therefore, selecting a polymer that can be easily dissolved, such as polyvinyl alcohol (PVOH), is preferred. In addition, the selection of PVOH as the polymer matrix in this study is attributed to its inherent properties, including its excellent biodegradability, film-forming capability, gas barrier performance, water solubility, adhesion, and toughness. These characteristics make PVOH suitable for a wide range of applications, particularly in biomedical fields [12]. Bee et al. [13] had reported that the addition of 0.5 phr CNTs in PVOH provided a better reinforcing effect than the addition of 1 phr CNTs. In another study conducted by Bee et al. [14], the authors observed that the elongation at break and tensile strength of PVOH nanocomposites gradually increased up to 54% with the addition of 0.2 to 0.3 phr of CNTs. Based on these findings, the CNT loading selected for the present study was in the range of 0.1 to 0.5 phr.

To address economic and environmental concerns, waste materials such as bagasse, eggshells, fly ash, and paper sludge have been incorporated into polymer matrices to produce new composite materials with enhanced functionalities [15]. For example, eggshell waste has been successfully utilized as an organic filler to replace the mineral calcium carbonate in bio-compounds due to its low density, non-abrasiveness, renewability, high filling levels, and cost-effectiveness [11]. Eggshells contain 94% calcium carbonate, 1% magnesium carbonate, 1% calcium phosphate and 4% organic matter [9]. Calcium carbonate is the major constituent of eggshell that can act as an inert filler, increasing the mechanical properties of materials. When comparing the density of calcium carbonate extracted from eggshells and mineral calcium carbonate, the density of mineral calcium carbonate is 0.467 g/cm^3^. It is slightly higher than that obtained from eggshells, which has density of 0.424 g/cm^3^. Using calcium carbonate extracted from eggshells instead of commercial calcium carbonate could increase the crystallinity of the composites [16]. This characteristic allows eggshells to become an excellent candidate for bulk quantity, lightweight, inexpensive and low load-bearing composite application. The significant quantity of eggshell waste generated daily poses environmental and health risks when improperly disposed of. While some eggshells are discarded in landfills, certain sites refuse to accept them due to the attached membrane, which attracts pests [17]. The increased production of chicken eggs in Asia has corresponded to a rise in the disposal of chicken eggshell waste. In pursuit of sustainable development goals, eggshell waste can be recycled, reused or transformed into valuable products. While the conventional method of disposing of eggshell waste is through landfill, this approach may inadvertently attract worms into the soil, potentially affecting its pH levels. To address this issue, there has been a growing interest in exploring alternative uses for eggshell waste. Notably, research indicates that eggshells can serve as biomaterials in therapeutic and medical applications owing to the presence of active compounds within them [18]. Beyond their environmental benefits, eggshell-derived materials have demonstrated significant potential as biomaterials for medical and therapeutic applications. Numerous studies have reported that both raw and calcined eggshell-derived calcium compounds exhibit excellent biocompatibility, bioactivity, and osteoconductive behaviour, supporting cell adhesion, proliferation, and bone tissue regeneration in both in vitro and in vivo studies [18]. Calcination effectively removes organic components and converts calcium carbonate into more bioactive calcium phases, such as calcium oxide or thermally modified calcium carbonate, which have been shown to be non-toxic and compatible with biological systems [18]. Before they can be applied for biomedical purposes, such as in bone implants, it is essential to evaluate the mechanical, thermal, and physical properties of these biomaterials. In the present study, eggshell waste was subjected to a calcination process to eliminate residual proteins and enhance its purity prior to its incorporation into the polymer matrix.

In this study, the eggshell waste was subjected to the calcination process to get rid of the protein content before it was incorporated into the polymer matrix. Bee et al. [4] observed that the incorporation of 1 phr to 3 phr calcined eggshell significantly improved the properties of PVOH blends. This study investigated the effects of incorporating calcined eggshell into polyvinyl alcohol (PVOH) loaded with carbon nanotubes (CNTs) to improve the physio-mechanical and thermal properties of PVOH blends [18]. Furthermore, the effect that adding CNTs has on the physical, mechanical and thermal properties of PVOH blends has also been investigated.

## 2. Materials and Methodology

### 2.1. Materials

**Figure 1:**
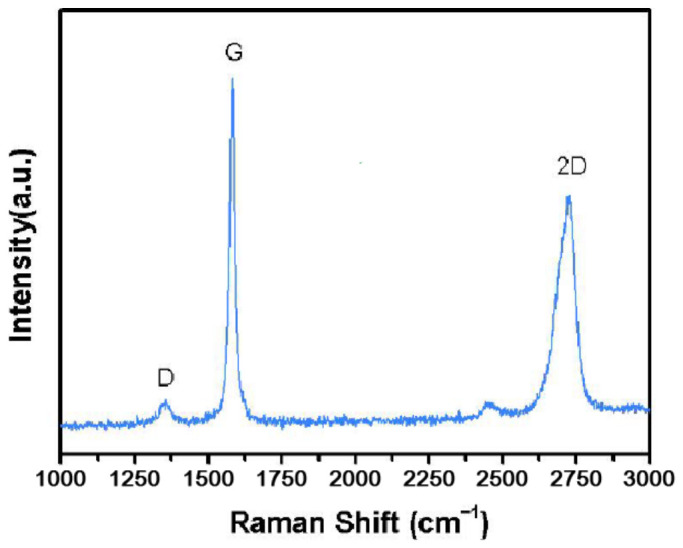
Raman spectrum of carbon nanotubes (CNTs) with a product code of XF022-1 (at wavelength of 785 nm) purchased from Nanjing XFNANO Materials Tech Co., Ltd.

Fully hydrolyzed PVOH resin was purchased from Sekisui Specialty Chemicals America, LLC., Dallas, CA, USA. The PVOH resin had a grade of 103 with characteristic behaviour of hydrolysis 98.4 mole % and 4% solution viscosity at 20 °C and 4.00 cP. Carbon nanotubes (CNTs, product code XF022-1) were purchased from XFNANO Materials Tech Co., Ltd., Nanjing, China and the Raman spectrum of this grade of CNTs (recorded at a wavelength of 785 nm) was provided by the manufacturer as shown in Figure 1. Based on the manufacturer’s specifications, the nanotubes have an outer diameter of >50 nm, an inner diameter in the range of 5–15 nm and CNTs lengths varying from 10 to 20 µm. The special surface area of the CNTs is approximately 40 m^2^/g. The material exhibits a carbon purity greater than 98% and a specific surface area of approximately 220 m^2^ g^−1^. These MWCNTs were synthesized using the chemical vapour deposition (CVD) method. Fully hydrolyzed PVOH resin was used as polymer base in this work. Multi-walled carbon nanotubes (CNTs) were used as reinforcing filler in this work. Lastly, the chicken eggshell wastes were collected from a bakery shop located at Sungai Long, Selangor, Malaysia.

### 2.2. Samples Preparation

#### 2.2.1. Preparation of Calcined Eggshell

The eggshell waste was collected from a bakery shop situated in Sungai Long, Selangor, Malaysia. It was meticulously washed with tap water to eliminate any dirt and egg white and yolk residues adhering on the eggshells. Additionally, the membrane layer sticked on the inner surface of the eggshells was carefully removed. After that, the cleaned eggshells were boiled and cooked in a 1000 mL beaker using a hot plate for a duration of 8 h to eliminate the presence of organic materials. Following this, the cooked eggshells underwent a drying process in a vacuum oven at a heating temperature of 60 °C for a duration of 20 h. These dried eggshells were then placed into a sealed bag and stored in a desiccator until the heat calcination process took place. Before proceeding with calcination, the dried eggshells were crushed into smaller particles using a crusher. The heat calcination process was performed in a furnace at a heating temperature of 900 °C for three hours of combustion. Finally, the calcined eggshells were further ground into a finer powder using a porcelain mortar. The prepared calcined eggshell powder was observed under SEM and the particles were approximately 250 µm in size.

#### 2.2.2. Preparation of Calcined Eggshell/PVOH/CNTs Composites

All the calcined eggshell/CNT-containing PVOH blends were prepared with 100 phr of PVOH resin and calcined eggshell with a loading level of 1 phr or 3 phr and CNTs with a loading level of 0.1 phr, 0.2 phr, 0.3 phr, 0.4 phr or 0.5 phr (phr is equivalent to parts per hundred resin). Firstly, 20 g of PVOH resin in powder form was weighed and dissolved in 20 mL of hot distilled water in a 1000 mL beaker using a water bath maintained at an approximate temperature of 97 ± 2 °C. In order to ensure complete dissolution of all PVOH particles, a motor-driven stirrer (a Cantilevel Type Constant Speed Electric Stirrer, model LG-Gz-20) was employed to agitate the solution thoroughly. Subsequently, calcined eggshell (0.2 g and 0.6 g, respectively) and carbon nanotubes (CNTs) (0.02 g, 0.04 g, 0.06 g, 0.08 g and 0.10 g, respectively) were weighed accordingly. After that, the weighed calcined eggshell and CNT were added into the PVOH solution and further mixed for 30 min to ensure complete homogenization. The resulting mixture solution was then poured into a plastic Petri dish to form a film, which was subsequently dried in a vacuum oven at 65 °C. Finally, the dried samples were placed in sealed plastic bags and stored in a desiccator at room temperature.

### 2.3. Characterization Testing

#### 2.3.1. X-Ray Diffraction (XRD) Test

A Shimadzu XRD 6000 X-ray Diffractometer (manufactured by Shimadzu Corporation based in Kyoto, Japan) was used to observe and investigate the crystal structure of the samples of PVOH nanocomposites containing calcined eggshell-CNTs. A Cu-K radiation generator with a 1.542 nm wavelength was used to determine the XRD spectra of all the samples. The rate of scanning of the generator was set to 1.2° per minute with a scattering angle (2θ) range of 0° to 40°. A 30 mA current and 40 kV voltage were used in this test. The d-spacing, d, of crystallite for deflection peak A was calculated using Bragg’s equation [19] as shown in Equation (1). The interchain separation, R, for crystallite deflection peak A was calculated by using the Klug and Alexander equation as listed in Equation (2). The crystallite size, L, for crystallite deflection peak A in the polymer matrix was determined using the Scherrer equation as shown in Equation (3).

(1) $$d=\frac{\lambda }{2\;\sin\theta }$$

(2) $$R=\frac{5\lambda }{8\sin\theta }$$

(3) $$L=\frac{K\lambda }{\beta \cos\theta }$$

where K is Scherrer constant, β is the full width at half maximum (FWHM) of the deflection peak, λ is 1.542 Å and θ is the Bragg angle in radians.

#### 2.3.2. Tensile Test

The tensile test was conducted using an Instron universal testing machine (Model 5848) (manufactured by Instron in Norwood, MA, USA) in accordance with ASTM D882 [20]. The cast samples were prepared with standardized dimensions of 1.5 mm thickness, 30 mm gauge length, and 3 mm width. For each formulation, five specimens were prepared and tested to ensure reproducibility. The cut specimens were mounted between the grips and then subjected to uniaxial tensile loading at a constant crosshead speed of 50 mm/min until the failure of the specimen. Then, the tensile strength, Young’s modulus, and elongation at break were determined from the resulting stress–strain curves, and the reported values represent the average of five measurements.

#### 2.3.3. Fourier Transform Infrared Spectroscopy (FTIR) Analysis

Fourier transform infrared (FTIR) analysis was employed to identify the presence of chemical groups in the samples by utilizing the Nicolet IS10-FTIR Spectrometer. Prior to sample placement, the surface of the machine was cleaned with alcohol. The samples were positioned precisely at the centre of the diamond crystal on the ATR plate. The infrared (IR) beam with a diameter of 0.1 mm was emitted by the instrument and directed to the centre of the diamond. Pressure was then exerted on the sample to facilitate scanning. The ATR press was gradually lowered, and the dial was rotated until an audible click was heard. The scanning rate for the test was configured within the range of 4000 cm^−1^ to 400 cm^−1^. Results were acquired by gradually lowering the press and rotating the dial until an audible click sound was detected.

#### 2.3.4. Scanning Electron Microscopy Analysis (SEM)

The surface morphology of both the calcined eggshell and film cast samples was examined using a Hitachi S3400 N Scanning Electron Microscopy (SEM) machine. Prior to SEM scanning, the fractured surface of each sample was fragmented into smaller pieces. These cut samples were affixed onto a specimen holder using carbon tape, ensuring that the fracture surface faced upward. Subsequently, an EMITECH SC7620 Sputter Coater was employed to apply a layer of platinum and silver coated onto the surface of the mounted samples. The coated samples were then loaded inside the chamber of the SEM machine and subjected to SEM scanning with a 30 kV electron beam voltage. The SEM machine captured the high-resolution images of the coated samples at magnification of 800 times. Additionally, Energy-Dispersive X-ray (EDX) analysis was utilized to determine the elemental composition of the calcined eggshell powder.

#### 2.3.5. Differential Scanning Calorimetry (DSC) Test

The thermal analysis of the samples of PVOH nanocomposites was carried out using a Mettler Toledo DSC823 differential scanning calorimeter (DSC). The samples of PVOH nanocomposites were weighed and placed in standard aluminum pans. On the other hand, a sealed empty aluminum pan was used to serve as the reference. Before the scanning process commenced, nitrogen gas was purged into the combustion column at a flow rate of 20 mL/min for 5 min to eliminate the presence of air. The DSC analysis was conducted over a temperature range of 25 °C to 240 °C, with a heating rate of 20 °C/min.

## 3. Results and Discussion

### 3.1. Energy Spectra X-Ray (EDX) Analysis of Calcined Eggshell

**Figure 2:**
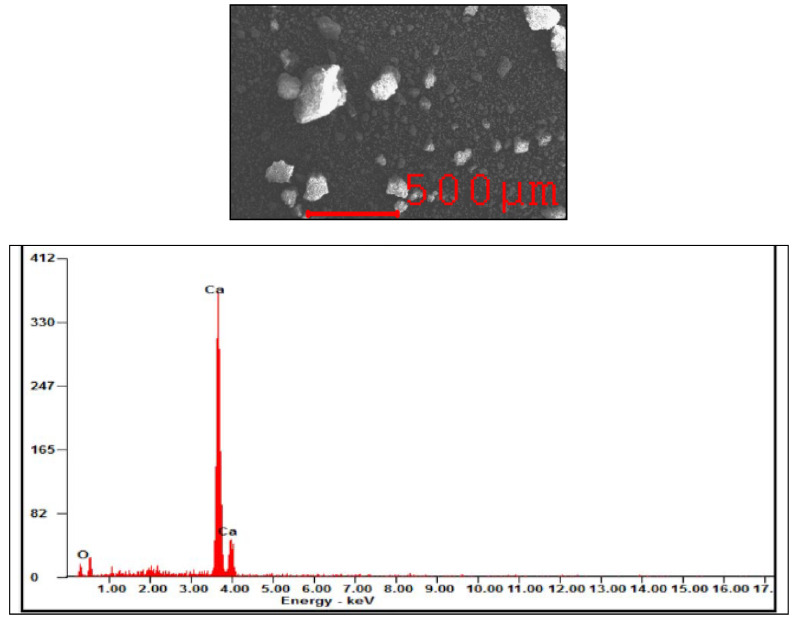
Energy spectra X-ray analysis result of calcined eggshell.

The EDX analysis of calcined eggshell, as depicted in Figure 2, was utilized to determine the elemental composition of the calcined eggshell. Calcium was identified as the predominant element within the range of 3.5 keV to 4.1 keV, while oxide was observed within the range of 0 keV to 1 keV, as evident from Figure 2. This suggests that the calcination of chicken eggshell effectively transformed the calcium carbonate into calcium oxide. During calcination, the primary constituent of eggshell waste calcium carbonate had been converted into calcium oxide, accompanied by the release of carbon dioxide, as illustrated in Equation (4) [18]. This is attributed to the fact that the raw chicken eggshell waste mainly consisted of calcium carbonate (CaCO_3_) and the calcination of CaCO_3_ at high temperature generated calcium oxide (CaO) by releasing carbon dioxide gas (CO_2_) as shown in Equation (4).

(4) $$CaCO_{3}\to CO_{2}+CaO$$

### 3.2. Characterization of PVOH and PVOH/MMT Blends

#### 3.2.1. XRD Analysis

**Figure 3:**
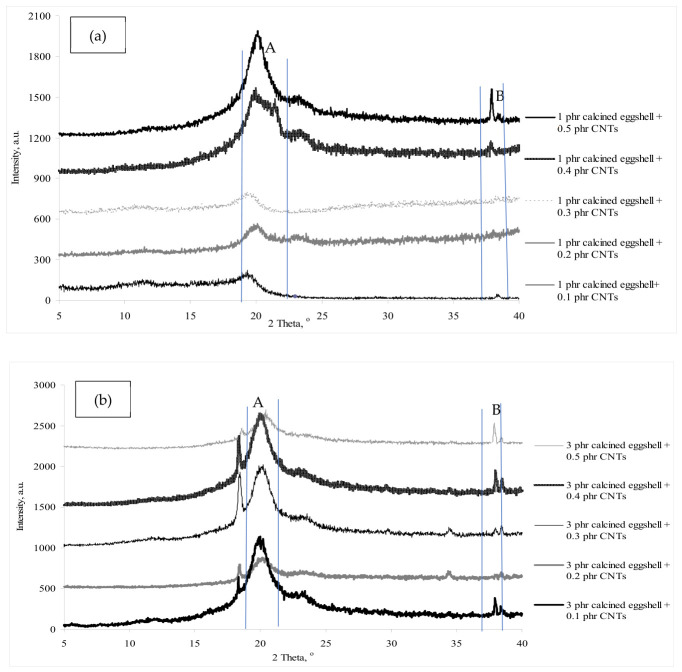
(**a**) The 1 phr calcined eggshell-containing PVOH blends with an increasing loading level of CNTs and (**b**) 3 phr calcined eggshell-containing PVOH blends with an increasing loading level of CNTs.

Figure 3a,b depict the XRD curves of all calcined eggshell-CNT/PVOH blends with increasing loading levels of CNT and calcined eggshell. Crystallite size represents the average dimension of ordered domains within a material that diffracts X-rays coherently and is commonly estimated using the Scherrer equation [19]. This parameter reflects the size of individual crystalline domains. In contrast, crystallinity describes the proportion of the material that exists in a crystalline state relative to the amorphous phase [19]. The degree of crystallinity is determined from the ratio of the integrated area of crystalline peaks to the total area of both crystalline and amorphous components in the XRD pattern. Notably, a significant deflection peak A is evident within the 19° to 21° range across the XRD curves of all calcined eggshell-CNT/PVOH blends, with the broad shape of the deflection peak indicating the appearance of a small crystallite size. The presence of this broad deflection peak in these curves suggests the presence of partial crystallites dominated by an amorphous phase [19]. Upon closer examination of Figure 3a,b, it is apparent that the deflection peak of the 3 phr calcined eggshell-containing CNT/PVOH blends exhibits a considerably sharper profile compared to the 1 phr calcined eggshell-containing CNT/PVOH blends. This sharpening effect indicates an increase in the crystallite size of deflection peak A, as corroborated by Table 1, which illustrates that higher calcined eggshell loading leads to larger crystallite sizes [21]. The addition of calcined eggshell influences the crystallite size of the deflection peak by effectively interacting with the small crystallites of calcined eggshell within the PVOH matrix [22,23].

Conversely, the addition of CNT to calcined eggshell–PVOH blends has reduced the crystallite size of the deflection peaks. This reduction may stem from the diminished interaction between the small crystallites of CNT and the crystallites of the deflection peak in the PVOH matrix [24,25], resulting in a restructuring of the crystallite structure within the PVOH matrix [26]. Consequently, this restructuring contributes to lower crystallinity, as evidenced in Table 1. For a constant 3 phr calcined eggshell loading, an increase in the amount of CNT in the PVOH matrix up to 0.5 phr led to an increase in crystallinity to 14.841%, as shown in Table 1. Higher CNT concentrations tend to promote higher crystallinity due to the agglomeration of CNTs resulting from non-homogeneous dispersion. In addition, the increase in crystallinity may also contribute to the formation of more ordered chains arrangement due to the good interaction of the CNTs and PVOH matrix [14].

Furthermore, examining Figure 3a,b, reveals deflection peak B at the 2θ range of 37° to 40° range with an increase in calcined eggshell loading from 1 phr to 3 phr. The sharpening effect observed in the deflection peak may be attributed to the agglomeration and uneven dispersion of calcined eggshell particles. However, when comparing 1 phr calcined eggshell with 0.5 phr CNT loading to 3 phr calcined eggshell with 0.5 phr CNT loading, the crystallite size decreased from 152 Å to 143 Å. This suggests that the addition of calcined eggshell to PVOH blends may disrupt the highly ordered chain structure of the deflection peak by reducing the interaction between existing deflection peak crystallites and newly formed crystallites [24,26], thereby reducing the crystallite size of the deflection peak. This finding aligns with the calculated crystallite size of deflection as shown in Table 1. Furthermore, Table 1 reveals that adding calcined eggshells particles and CNT to the PVOH matrix has minimal effect on the d-spacing and interchain separation of deflection peaks A and B, indicating that the presence of calcined eggshells and CNT particles in the PVOH matrix does not significantly affect the compactness of these deflection peaks [27].

#### 3.2.2. Tensile Properties

##### Tensile Strength

**Figure 4:**
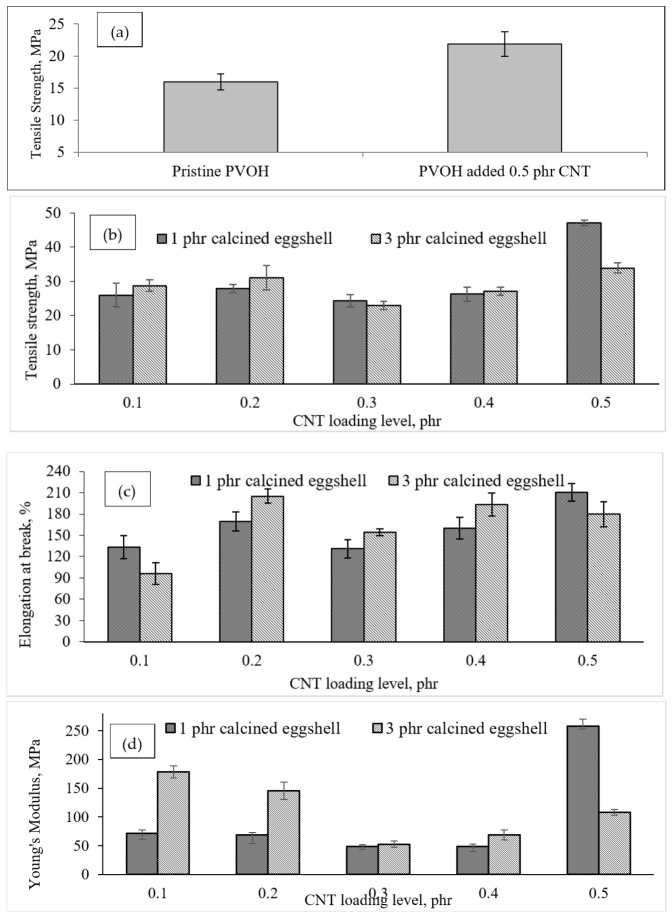
(**a**) Tensile strength of pristine PVOH, 0.5 phr containing PVOH blend [28] and the effect of increasing CNTs loading level on (**b**) tensile strength, (**c**) elongation at break and (**d**) Young’s modulus of all calcined eggshell/PVOH/CNTs blends.

The tensile strength of pristine PVOH was determined to be 15.98 ± 1.22 MPa as shown in Figure 4a. According to a study by Bee et al. [28], carbon nanotubes (CNTs) of grade XF022-1 were incorporated into PVOH. Their results showed that adding 0.5 phr of CNTs significantly increased the tensile strength of PVOH to 21.85 ± 1.89 MPa (as depicted in Figure 4a). However, further increasing the CNT content from 0.5 phr to 1 phr resulted in a reduction in tensile strength. According to these results [28], the addition of 0.5 phr CNTs could provide an optimum improvement in tensile strength compared to 1 phr of CNTs containing PVOH blends. Based on the findings of Bee et al. [28], the 0.5 phr CNTs were selected to form hybrid fillers in PVOH composites in this study. By comparing the results of this study with the results reported by Bee et al. [28] for PVOH blends containing a single filler of CNTs, it was found that the incorporation of hybrid fillers consisting of CNTs and calcined eggshell resulted in higher tensile strength than PVOH blends filled with CNTs alone, as shown in Figure 4b.

According to Figure 4b, when comparing the tensile strength of samples containing 1 phr and 3 phr of calcined eggshell with a low amount of carbon nanotubes (0.1 phr), the tensile strength of the sample containing 3 phr calcined eggshell was observed to be 5.902% higher than the sample containing 1 phr calcined eggshell. This suggests that a higher concentration of calcined eggshell led to an improvement in the interaction between the calcined eggshell and the PVOH matrix. In essence, transferring the applied tensile stress from the PVOH matrix to the calcined eggshell particles was found to be more effective [29]. Additionally, the hydrogen bonds linking the calcined eggshell particles to the PVOH matrix contributed to an effective reinforcing effect, thus enhancing the interaction between the calcined eggshell and the PVOH matrix. Consequently, the sample containing 3 phr calcined eggshell required an additional application of tensile stress compared to the sample containing 1 phr calcined eggshell. A similar trend was observed when the amount of carbon nanotubes increased to 0.2 phr. Observations from Figure 4b indicate a subtle contrast between the samples containing 1 phr and 3 phr of calcined eggshell at 0.3 phr and 0.5 phr of carbon nanotubes (CNTs). Notably, the tensile strength of the sample containing 1 phr calcined eggshell surpassed that of the sample with 3 phr CE for both 0.3 phr and 0.5 phr CNTs. This discrepancy can be attributed to the a higher loading level of calcined eggshell tending to form larger agglomerated particles due to uneven distribution during the solution mixing process. The incorporation of calcined eggshell with quantities of up to 3 phr has significantly exacerbated the agglomeration effect of calcined eggshell particles within the PVOH matrix compared to the 1 phr calcined eggshell. It is noteworthy that minimal agglomeration of calcined eggshell could potentially enhance tensile strength, as agglomerated particles may disrupt alignment within the PVOH matrix [30,31]. However, Figure 4b also reveals that the tensile strength of the sample containing 3 phr calcined eggshell was 0.259 MPa higher than that of the sample containing 1 phr calcined eggshell at 0.4 phr CNT. This improvement can be attributed to the effective dispersion of calcined eggshell particles within the PVOH matrix.

The trend observed indicates that as the amount of carbon nanotubes (CNTs) increases in both 1 phr and 3 phr calcined eggshell-containing PVOH blends from 0.1 phr to 0.3 phr, the tensile strength of both 1 phr and 3 phr calcined eggshell-containing PVOH blends was also observed to increase. The occurrence of this phenomenon is mainly because the CNT particles serve as a reinforcing agent within the composite matrix, enhancing its mechanical strength. Furthermore, the crystallinity of both calcined eggshell-containing PVOH blends was found to be higher. This because the presence of more crystallite structures could provide a better reinforcing effect within the PVOH matrix. However, a decrease in tensile strength was noted in the 0.3 phr CNT composition compared to the 0.2 phr CNT composition. This reduction can be attributed to the tendency of CNT to agglomerate within the PVOH matrix upon addition to the blends. The inadequate interaction between CNT and the PVOH matrix leads to the agglomeration of CNT particles, resulting in poor interfacial adhesion between CNT and the PVOH matrix. Consequently, the transfer of stress from the PVOH matrix to the agglomerated side decreases during straining [28,32].

##### Elongation at Break

Figure 4d illustrates the impact of increasing the amount of carbon nanotubes (CNTs) on the elongation at break of all calcined eggshell-containing PVOH blends. The results obtained for elongation at break have exhibited a similar pattern to the results of tensile strength. Specifically, the elongation-at-break value of both calcined eggshell-containing samples increased from 0.1 phr CNT to 0.2 phr CNT and then decreased at 0.3 phr CNT. However, the elongation at break was found to rise again after surpassing 0.3 phr CNT. The elongation at break for all blends remained within the range of 120% to 210% for 1 phr CE and 80% to 190% for 3 phr calcined eggshell, respectively.

Upon closer examination of Figure 4d, it can be observed that 1 phr calcined eggshell exhibited a higher elongation-at-break value than 3 phr calcined eggshell-containing samples for CNT loadings of 0.1 phr, 0.2 phr, and 0.5 phr. This disparity arises due to the fact that the presence of lower amount of calcined eggshell would act as a lubricant within the PVOH matrix when subjected to straining stress. A small quantity of calcined eggshell could enhance the alignment of CNTs within the PVOH chain arrangement, thereby promoting the slippage effect of PVOH chains during extension [33]. However, exceeding the critical limit of calcined eggshell content leads to a reduction in the matrix continuity of the PVOH matrix. Additionally, the agglomeration of calcined eggshell particles within the PVOH matrix, resulting from uneven dispersion, hinders the flow of polymer chains in the PVOH matrix during straining [26]. Consequently, the ability of the PVOH matrix to elongate at high calcined eggshell-loading levels is significantly diminished. In Figure 4c, pristine PVOH can be observed to pose the highest value of elongation-at-break compared to all calcined eggshell-containing PVOH blends. This is because the pristine PVOH has non-restricted polymer chains, meaning that the polymer chains can slip freely on each other when subjected to straining [29,32]. The elongation-at-break value reached its minimum when the concentration of calcined eggshell was 1 phr. As more calcined eggshell particles were added into the polymer matrix of PVOH composite, increased agglomeration occurred due to inadequate dispersion. Consequently, this hindered the effective bonding between calcined eggshell particles and the PVOH matrix, resulting in decreased interfacial adhesion. This diminished adhesion caused the PVOH matrix to exhibit brittle behaviour, thereby reducing the elongation value of the composite [27]. However, with further additions of calcined eggshells, the elongation value increased again, as evidenced by Figure 4d. It is plausible that at this loading of PVOH, the interlayer gallery spacing of calcined eggshell particles may have been filled, allowing free PVOH to encapsulate calcined eggshell particles or agglomerates, thereby acting as a plasticizer [26]. This plasticizing effect contributed to the subsequent increase in elongation-at-break [34,35,36,37,38].

From Figure 4d, it is evident that Young’s modulus demonstrates a declining trend with increasing CNT content up to 0.3 phr. This trend is observed to be consistent across both 1 phr and 3 phr calcined eggshell concentrations, indicating that higher amounts of CNT particles do not enhance the stiffness of the PVOH blends. The reduction in stiffness of the blends can be attributed to the inadequate interaction between the filler and the PVOH matrix. The aggregation of particles plays a significant role in causing poor dispersion of the filler within the PVOH matrix. This phenomenon can be elucidated by the formation of weak hydrogen bonds between the filler and the PVOH matrix, leading to phase separation that disrupts the continuity of the PVOH matrix within the CNT interlayer galleries, consequently weakening the mechanical properties of the composite [36]. This study suggests that at higher filler concentrations, the adhesion quality between the reinforcing filler and the PVOH matrix, as well as the dispersion level of the filler within the PVOH matrix, were notably deficient. As a result, an increase in the amount of CNT could increase the Young’s modulus of the PVOH matrix. According to Ismail and Shaari [27], a stronger interaction between filler and polymer matrix diminishes elasticity and impedes the movement of polymer chains, resulting in blends with tougher and more rigid properties.

#### 3.2.3. FTIR Analysis

**Figure 5:**
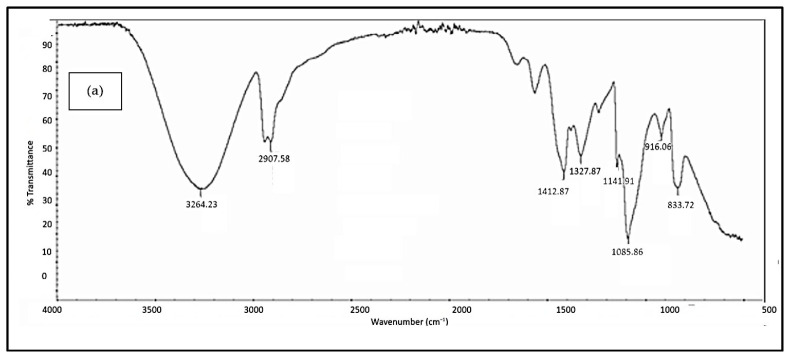

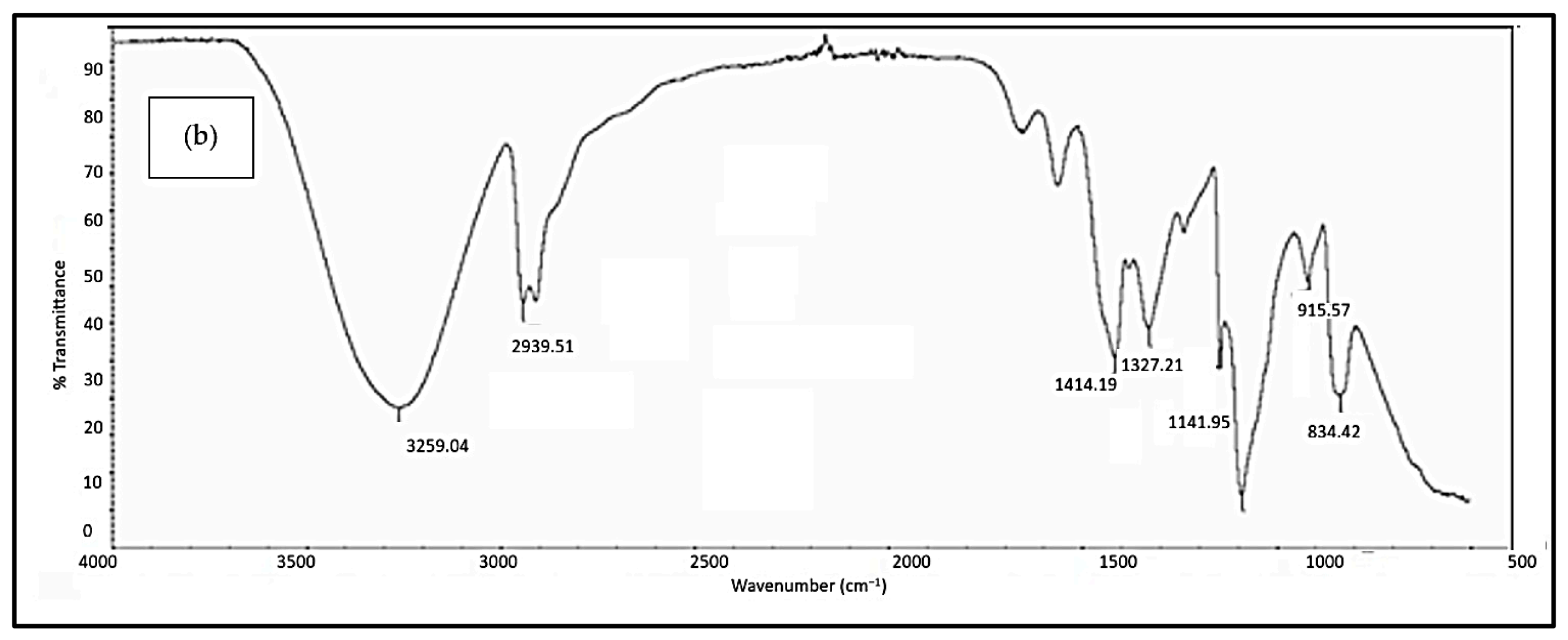
FTIR spectrum of 1 phr calcined eggshell/PVOH composites containing (**a**) 0.1 phr CNTs and (**b**) 0.2 phr CNTs.

**Figure 6:**
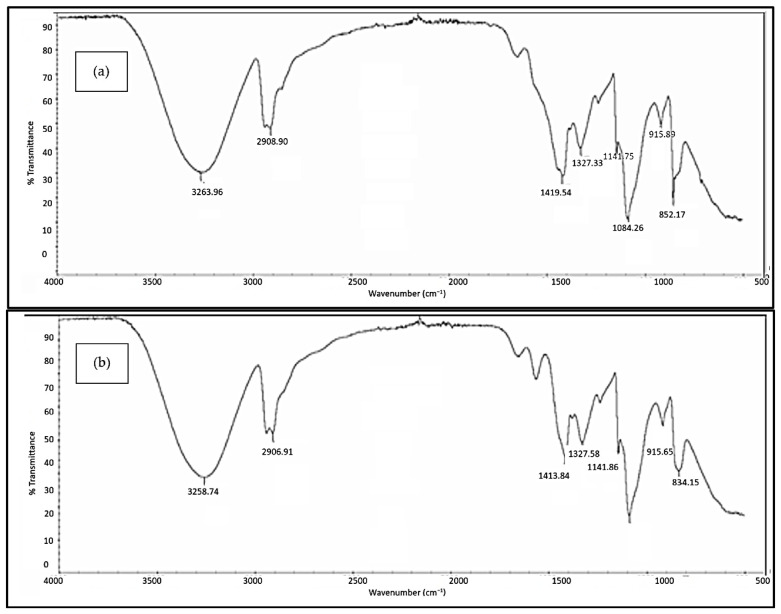
FTIR spectrum of 1 phr calcined eggshell/PVOH composites containing (**a**) 0.3 phr CNTs and (**b**) 0.5 phr CNTs.

**Figure 7:**
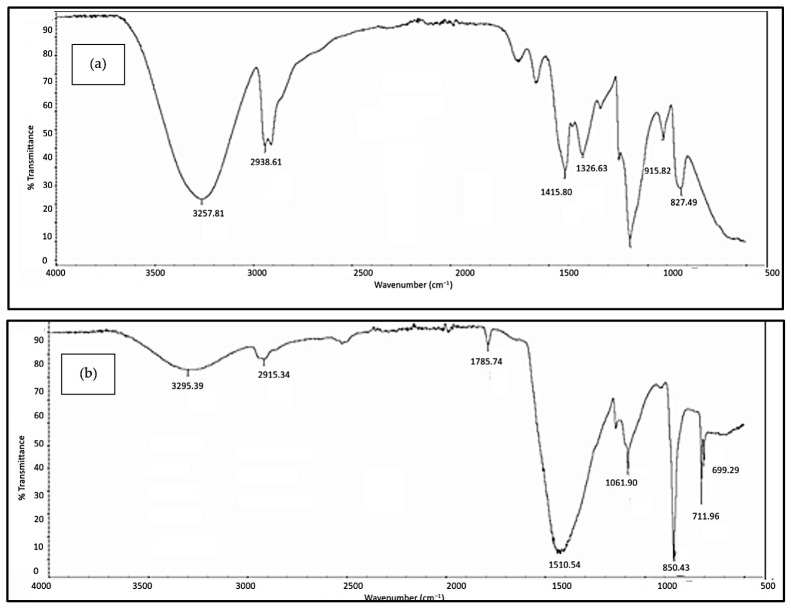
FTIR spectrum of (**a**) 1 phr calcined eggshell/PVOH composites containing 0.5 phr CNTs and (**b**) 3 phr calcined eggshell/PVOH composites containing 0.1 phr CNTs.

**Figure 8:**
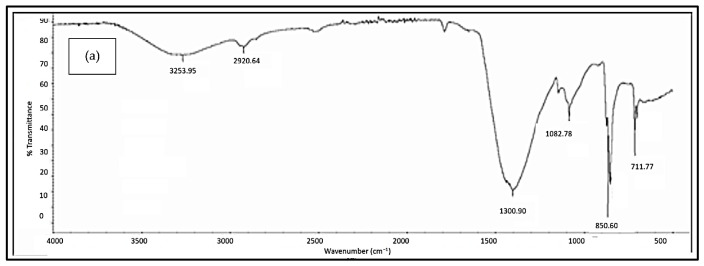

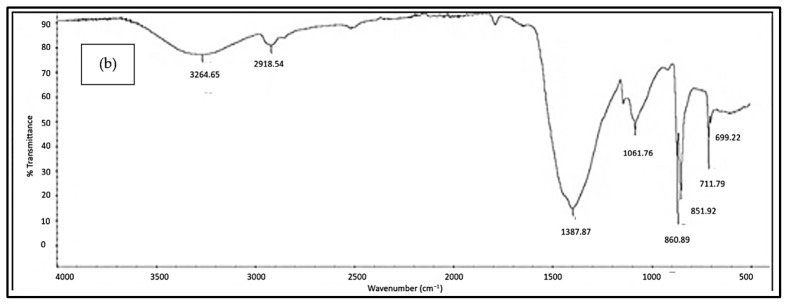
FTIR spectrum of 3 phr calcined eggshell/PVOH composites added with (**a**) 0.2 phr CNTs and (**b**) 0.3 phr CNTs.

**Figure 9:**
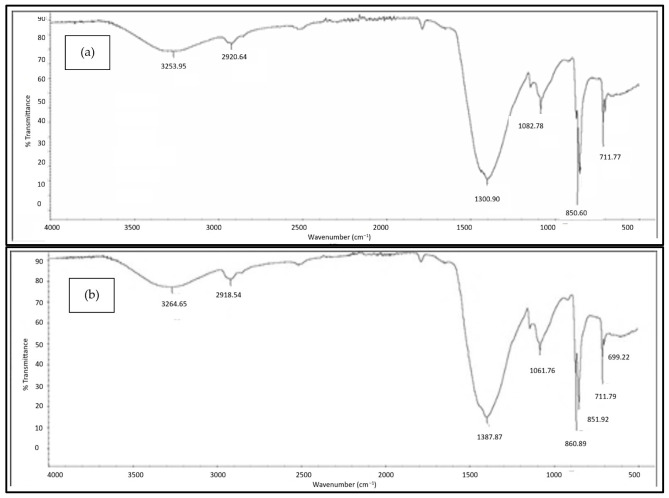
FTIR spectrum of 3 phr calcined eggshell/PVOH composites containing (**a**) 0.4 phr CNTs and (**b**) 0.5 phr CNTs.

Figure 5, Figure 6, Figure 7, Figure 8 and Figure 9 illustrate the infrared spectrum of PVOH blends containing increasing loading levels of CNT and calcined eggshell. Notably, a distinct peak is found to appear within the band of 3250 cm^−1^ to 3300 cm^−1^ in the FTIR spectrum, indicating the existence of hydroxyl group (-OH) stretching in the PVOH blends [25,39]. According to Table 2, the increase in calcined eggshell from 1 phr to a 3 phr loading level in CNT-PVOH blends was found to significantly increase the wavenumber of O-H bonding in the FTIR spectrum. This indicates that the higher amounts of calcined eggshell (CaO) in the polymer matrix of CNT-PVOH blends have improved the bonding strength of O-H bonds within the lattice structure. A higher loading level of calcined eggshell content indicates the existence of a high amount of CaO. This might be attributed to the higher-loading-level calcined eggshell content (CaO) tending to agglomerate together, reducing the effective interfacial adhesion effect between calcined eggshell particles and the PVOH matrix [37]. This could further cause the -OH groups in the PVOH matrix to prefer interacting among themselves compared to their interaction with CaO (calcined eggshell) particles at higher loading levels of calcined eggshell. Thus, the bonding strength of O-H bond significantly increased when the calcined eggshell increased from 1 phr to 3 phr [36]. In other words, the elevated calcined eggshell content in the PVOH matrix disrupted the hydrogen bonding between polymer chains.

On the other hand, Figure 5, Figure 6, Figure 7, Figure 8 and Figure 9 illustrate a rightward shift in the wavenumber of the O-H stretching band with increasing CNT content, leading to a decrease in wavenumber. This observation indicates that the presence of a higher amount of CNTs in a polymer matrix of a calcined eggshell/PVOH blend significantly improved the interaction between calcined eggshell and PVOH matrix. The higher amount of CNT particles could reduce the agglomeration of calcined eggshell particles. This can be explained by the fact that the reduction in the wavenumber of O-H groups is primarily attributed to the presence of stronger hydrogen bonding within the PVOH matrix [36,37]. Observations from Table 2 indicate a slightly lower wavenumber of O-H bonding in 0.3 phr CNTs compared to 0.4 phr CNTs, which could possibly be attributed to the higher CNTs content of up to 0.4 phr causing a loss of the advantage that CNT particles usually provide in terms of improving the interaction effect between calcined eggshell particles and the PVOH matrix.

On the other hand, the presence of a C-H stretching bond was observed to appear at the range of 2900 cm^−1^ to 2940 cm^−1^, as evident in Figure 5, Figure 6, Figure 7, Figure 8 and Figure 9. An analysis of Table 2 reveals an increase in C-H bonding as CNTs content increases. This phenomenon might be attributed to the hydrophobic nature of CNT particles, which tend to interact with the -CH bond rather than the -OH bond [4,36]. Consequently, a reverse trend between the wavenumbers of O-H and C-H bonds is observed as the CNT content increases. With increasing CNT loading, the inherently hydrophobic surface of the nanotubes shows a greater affinity for the non-polar C–H moieties of the polymer matrix than for polar O-H groups [40]. This preferential interaction alters the local molecular environment, resulting in noticeable changes in vibrational behaviour, including shifts in the wavenumbers of C-H and O-H. Specifically, the C–H bands exhibit an enhancement in intensity, while the O-H stretching bands shift because of weakened intra- and intermolecular hydrogen bonding. These spectral variations indicate that the incorporation of CNTs perturbs molecular vibrations and modifies local bonding environments, particularly at higher filler concentrations [41], where interactions between hydrophobic polymer segments and the CNT surface become more pronounced.

In addition, the O-H bending bond was observed to be on the FTIR spectrum within a wavenumber range between 1326 cm^−1^ and 1328 cm^−1^ for all samples of PVOH blends containing 1 phr of calcined eggshell, as depicted in Figure 5, Figure 6 and Figure 7a. However, this O-H bending bond was observed to be diminished as the calcined eggshell content increased, as evidenced in Figure 7b, Figure 8 and Figure 9. This disappearance is attributed to the strong interaction between calcined eggshell particles and the hydroxyl group in the PVOH matrix, which restricted the -OH bending and improved the molecular bonding strength within the CE-CNT/PVOH blends [36,38].

#### 3.2.4. Surface Morphologies Observation

**Figure 10:**
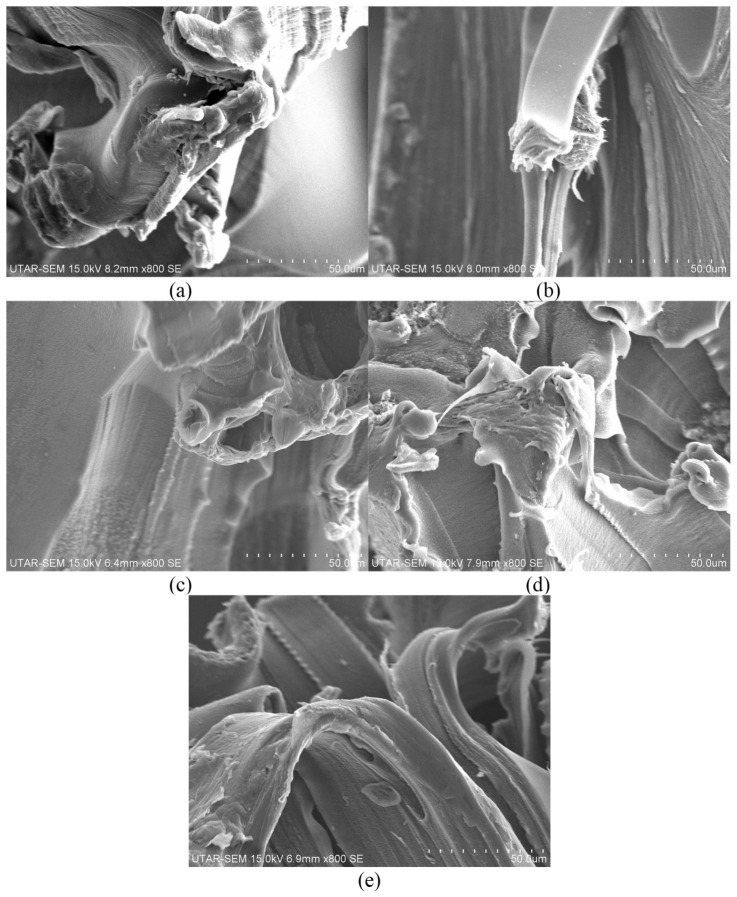
SEM micrographs of fractured surface for 1 phr calcined eggshell/PVOH blends containing (**a**) 0.1 phr CNTs, (**b**) 0.2 phr CNTs, (**c**) 0.3 phr CNTs, (**d**) 0.4 phr CNTs, and (**e**) 0.5 phr CNTs, respectively, magnified 800 times.

**Figure 11:**
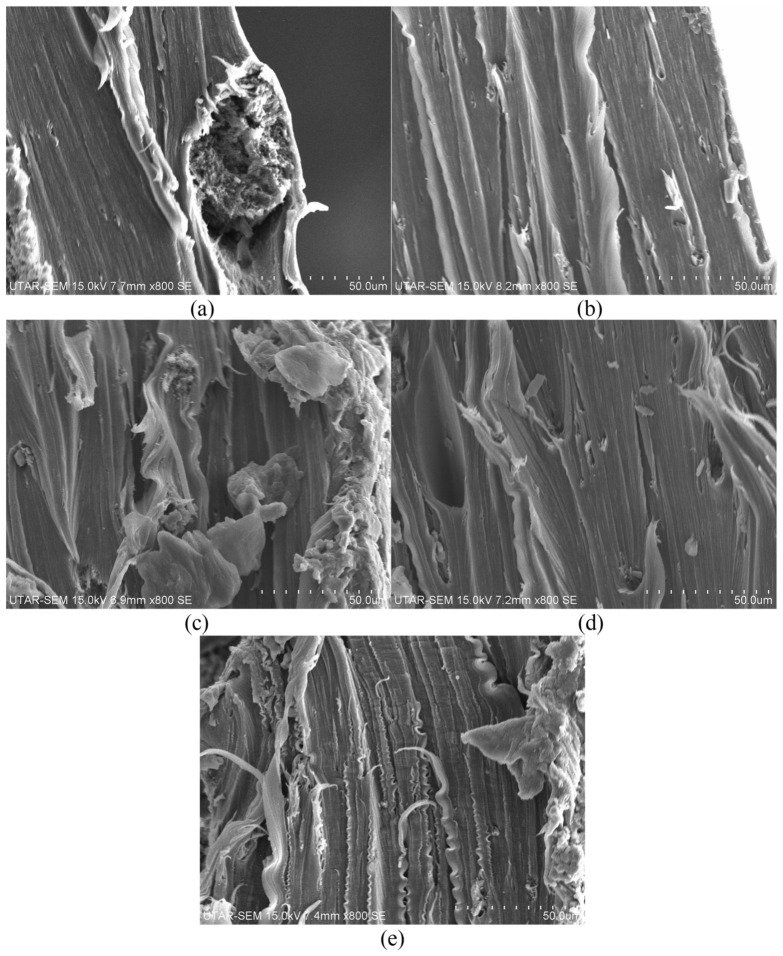
SEM micrographs of fractured surface for 3 phr calcined eggshell/PVOH blends added with (**a**) 0.1 phr CNTs, (**b**) 0.2 phr CNTs, (**c**) 0.3 phr CNTs, (**d**) 0.4 phr CNTs, and (**e**) 0.5 phr CNTs, respectively, magnified 800 times.

The impact of varying CNT content on the fracture surface characteristics of both 1 phr and 3 phr calcined eggshell/PVOH blends is depicted in Figure 10 and Figure 11. The inclusion of CNT in all calcined eggshell/PVOH blends led to an increase in the surface roughness and the clustering of CNT particles. Notably, Figure 10b shows a more pronounced formation of fibres compared to Figure 10a. The appearance of fibrous stems in the PVOH matrix is indicative of the high level of resistance to elongation when the sample is subjected to straining action. In addition, minimal CNT particle clustering was observed for the samples containing lower CNT concentrations of 0.1 phr and 0.2 phr. Conversely, for the samples containing higher concentrations of 0.3 phr and 0.4 phr, significant occurrence of particle agglomeration within the polymer matrix of calcined eggshell/PVOH blends was observed, as depicted in Figure 10. The agglomerated particles act as stress concentration points, potentially leading to reduced tensile strength and premature film failure. The observed fracture morphology of the nanocomposite aligns with the previously discussed tensile test results.

Figure 11 presents the SEM images illustrating the fracture surface morphologies of 3 phr calcined eggshell/PVOH blends containing varying amounts of CNT. By observing Figure 11a, a significant amount of particle agglomeration was observed to appear on the fractured surface of low-CNT-containing 3 phr calcined eggshell/PVOH blends. The existence of the particle’s agglomeration is most likely induced by the higher loading levels of calcined eggshell particles inside polymer matrix of PVOH blends. A comparison between Figure 11d,e reveals that the heightened surface roughness correlates with an increase in tensile strength. Additionally, Figure 11e displays a wavier line structure, potentially contributing to a slight enhancement in tensile strength. At a CNT loading of 0.5 phr, the increased roughness and tearing necessitated a higher load to break the films.

#### 3.2.5. Thermal Analysis

**Figure 12:**
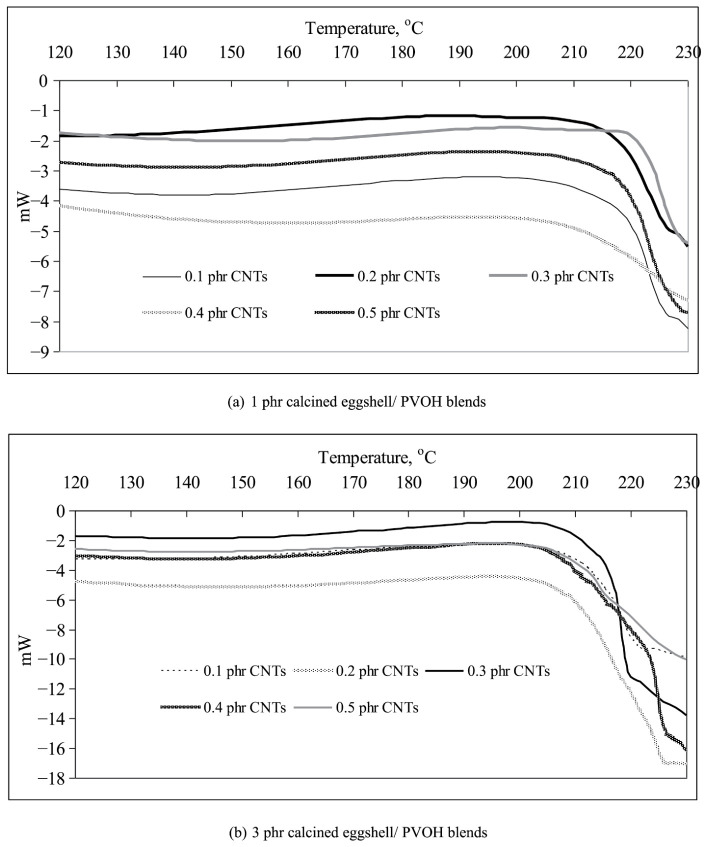
DSC thermograms of (**a**) 1 phr calcined eggshell/PVOH blends and (**b**) 3 phr calcined eggshell/PVOH blends containing an increasing loading level of CNTs.

Figure 12 displays the DSC thermograms for all CNT-PVOH blends with varying levels of calcined eggshell loading. A notable endothermic peak indicating the melting state is observed in the temperature range of 120 °C to 240 °C. Figure 12a,b show that the intensity and area of the endothermic peaks in all PVOH-calcined eggshell blends slightly increased with the rising CNT amounts up to 0.3 phr. The presence of intermolecular bonding in these blends can be investigated through the melting state of DSC endothermic peaks, where a larger peak area suggests a higher thermal energy requirement for polymer molecules to escape from the ordered crystallite structure [38]. Additionally, a broader melting peak area may indicate an enhanced arrangement of the crystallite structure due to the higher molecular weight. This observation is found to be consistent with the enthalpy of melting of all PVOH-calcined eggshell blends as summarized in Table 2. The increasing of CNT amounts up to 0.3 phr significantly increased the enthalpy of melting. This might be because the incorporation of CNT particles at loading levels below 0.3 phr appears to effectively interact with PVOH chains and calcined eggshell particles, thereby increasing intermolecular bonding within the polymer matrix of all CNT-containing PVOH-calcined eggshell blends [28]. Consequently, higher thermal energy is necessary to disrupt the intermolecular bonding between CNT, calcined eggshell particles, and the PVOH matrix. However, as the CNT loading level increases from 0.3 phr to 0.5 phr for all PVOH-calcined eggshell blends, the intensity and peak area of the endothermic peak noticeably decrease and broaden. By referring to Table 2, the enthalpy of melting of the PVOH-calcined PVOH severely reduced when the loading levels of CNT were higher than 0.3 phr. This reduction and broadening may be attributed to the severe agglomeration of CNT particles within the PVOH matrix at higher loading levels, disrupting the ordered chain arrangement structure [42]. Consequently, the thermal energy needed to overcome intermolecular bonding in the polymer matrix of PVOH-calcined eggshell blends with higher CNT amounts (>0.3 phr) was significantly reduced, further diminishing the intensity and area of the endothermic peak [43].

Table 2 shows that increasing the calcined eggshell loading level from 1 phr to 3 phr significantly increased the enthalpy of melting for all CNT-containing PVOH blends. This indicates that the presence of a higher amount of calcined eggshell could induce the required thermal energy to break the intermolecular bonding in the PVOH matrix [42,43]. This might be because the presence of a higher amount of calcined eggshell could interact well with PVOH macromolecular chains and enhance the strength of intermolecular bonding. Thus, the enthalpy of melting of the CNT-PVOH blends containing a higher amount of calcined eggshell also significantly increased the optimal melting temperature of all PVOH blends, with low levels of added calcined eggshell (1 phr) experiencing a slight increase from 217.6 °C to 219.9 °C as the CNTs content increased from 0.1 phr to 0.3 phr. This finding aligns with the observation in Figure 11a, in which the endothermic peaks shifted towards higher temperatures. The improved dispersion of CNT particles at lower concentrations (<0.3 phr) effectively promoted the intermolecular bonding in PVOH blends with low loading of calcined eggshell (1 phr). The well-dispersed low amounts of CNT particles interacted efficiently with the small quantity of calcined eggshell in the PVOH matrix, strengthening the intermolecular bonding. Consequently, more thermal energy is required to disrupt these bonds, especially hydrogen bonding interactions [39]. However, the optimal melting temperature of PVOH blends which contained higher CNT amounts (>0.3 phr) significantly increased with a further increase in calcined eggshell loading up to 3 phr, as indicated in Table 2. The presence of high levels of both CNT and calcined eggshell induced intermolecular bonding in the PVOH matrix, resulting in a slight elevation of the optimal melting temperature. This is attributed to the higher loading levels of calcined eggshell and CNT particles within the polymer matrix of PVOH blends, which substantially enhanced the heat capacity, necessitating higher kinetic energy and temperature to overcome the intermolecular bonding [41]. Consequently, the optimal melting temperature increased with the addition of higher levels of calcined eggshell.

## 4. Conclusions

In this study, eggshell waste underwent a calcination process in a furnace to convert it into usable calcium oxide, which was subsequently analyzed via EDX analysis. The incorporation of higher quantities of calcined eggshell significantly increased the tensile strength of all CNT-containing PVOH blends, whereas adding 0.5 phr of CNTs significantly increased the tensile strength of PVOH to 21.85 ± 1.89 MPa. This finding showcases the good interfacial adhesion effect between calcined eggshell and the PVOH matrix, which might be attributed to the hydrophilic behaviour of calcined eggshell particles and the PVOH matrix. However, the tensile strength and elongation-at-break of PVOH-calcined eggshell blends slightly decreased when the loading level of CNT increased from 0.2 phr to 0.3 phr. This was mainly due to the agglomeration of CNT particles in the PVOH-calcined eggshell matrix. XRD analysis revealed the presence of two deflection peaks. The sharpening effect on deflection peaks can be significantly observed for the CNT-containing PVOH blends containing 3 phr calcined eggshell, indicating that a higher amount of calcined eggshell could induce interaction between the calcined eggshell and PVOH matrix by expanding the size of crystallite through the convergence of less ordered structures to a more ordered arrangement. SEM depicted better dispersion of low amounts of calcined eggshell and CNTs in the PVOH matrix. Interestingly, DSC findings indicated that increasing the calcined eggshell loading level from 1 phr to 3 phr significantly increased the enthalpy of melting temperature of the CNT-PVOH blends. This indicates that a higher calcined eggshell loading level enhanced the heat capacity of all CNT-containing PVOH blends and required higher kinetic energy and temperature to overcome the intermolecular bonding. FTIR analysis revealed an increase in the -OH stretching band as the calcined eggshell loading level increased from 1 phr to 3 phr, indicating weakening hydrogen bonds within the PVOH matrix. Conversely, the bending of -OH disappeared with increasing calcined eggshell loading due to strong calcined eggshell–hydroxyl group interactions. Future works should focus on controlled modification of CNT surface chemistry—for example, via targeted functionalization—to more effectively tailor interactions with both polar and non-polar groups within polymer chains. Such approaches may promote improved CNT dispersion, stronger interfacial adhesion, and more consistent modulation of molecular vibrational behaviour as revealed by spectroscopic methods such as FTIR.

## Figures and Tables

**Table 1:** 2 Theta, crystallite size, d-spacing, interchain separation and crystallinity of crystallite structures of all calcined eggshell/PVOH blends with an increasing loading level of CNTs.

Loading Level of Calcined Eggshell, phr	Loading Level of CNTs, phr	2 Theta (2θ), °	Crystallite Size (L), Å	D-Spacing, Å	Interchain Separation (R), Å	Crystallinity, %
		Deflection Peak A				
1	0.1	19.05	42.04	4.65	5.82	15.35
1	0.2	19.89	57.26	4.46	5.57	13.52
1	0.3	19.31	51.73	4.59	5.74	15.26
1	0.4	19.88	51.58	4.46	5.58	11.34
1	0.5	20.10	49.11	4.41	5.52	11.49
3	0.1	19.92	51.13	4.45	5.56	10.96
3	0.2	20.18	50.26	4.39	5.49	13.59
3	0.3	20.08	50.13	4.42	5.52	12.64
3	0.4	20.06	48.49	4.42	5.53	12.79
3	0.5	20.30	48.20	4.37	5.46	14.84
		Deflection peak B				phr: Parts per hundred resin
1	0.1	-	-	-	-	
1	0.2	-	-	-	-	
1	0.3	-	-	-	-	
1	0.4	37.84	154.61	2.37	2.97	
1	0.5	37.98	152.55	2.37	2.96	
3	0.1	37.98	140.48	2.37	2.96	
3	0.2	38.33	132.93	2.35	2.93	
3	0.3	38.29	122.51	2.35	2.93	
3	0.4	38.04	131.95	2.36	2.95	
3	0.5	37.87	143.60	2.37	2.97	

**Table 2:** Wavenumber of O-H stretching bond and C-H stretching; enthalpy of the melting and melting temperature of all calcined eggshell/PVOH blends containing an increasing loading level of CNTs.

PVOH Blends Containing		Wavenumber, cm^−1^		Enthalpy of Melting (ΔHm), J/g	Melting Temperature, °C
Loading Level of Calcined Eggshell, Phr	Loading Level of CNTs, Phr	O-H Stretching	C-H Stretching		
1 phr	0.1	3264.23	2907.58	125.73	217.6
	0.2	3263.96	2908.90	378.43	217.7
	0.3	3256.85	2906.91	75.20	219.9
	0.4	3259.04	2939.51	150.46	207.0
	0.5	3257.81	2938.61	344.87	206.8
3 phr	0.1	3296.39	2915.34	465.60	216.0
	0.2	3279.68	2915.90	597.57	215.1
	0.3	3279.51	2918.43	525.39	215.5
	0.4	3263.95	2920.64	431.16	209.1
	0.5	3264.65	2918.54	462.40	209.0

## Data Availability

The raw data supporting the conclusions of this article will be made available by the authors on request.

## Abbreviations

CNT—carbon nanotube; PVOH—polyvinyl alcohol; XRD—X-ray diffraction; FTIR—Fourier transform infrared spectroscopy; SEM—scanning electron microscopy.
